# Supplementary material for: A partnership between the lipid scramblase XK and the lipid transfer protein VPS13A at the plasma membrane
Source: Proc Natl Acad Sci U S A. 2022 Aug 22;119(35):e2205425119. doi: 10.1073/pnas.2205425119 (PMC9436381; doi:10.1073/pnas.2205425119)
Supplement: Supplementary File [file pnas.2205425119.sapp.pdf]

Supplementary Information for

A partnership between the lipid scramblase XK and the lipid transfer protein VPS13A at the plasma membrane

Andrés Guillén-Samander[a,b,c,d], Yumei Wu[a,b,c,d], S. Sebastian Pineda[e,f,g], Francisco J. García[h,i], Julia N. Eisen[a,b,c,d], Marianna Leonzino[a,b,c,d,j,k], Berrak Ugur[a,b,c,d], Manolis Kellis[e,f,g], Myriam Heiman[h,i], and Pietro De Camilli[a,b,c,d,l]

[a]Department of Neuroscience, Yale University School of Medicine, New Haven, CT 06510
[b]Department of Cell Biology, Yale University School of Medicine, New Haven, CT 06510
[c]HHMI, Yale University School of Medicine, New Haven, CT 06510
[d]Program in Cellular Neuroscience, Neurodegeneration, and Repair, Yale University School of Medicine, New Haven, CT 06510
[e]Department of Electrical Engineering and Computer Science, Massachusetts Institute of Technology, Cambridge, MA 02142
[f]Computer Science and Artificial Intelligence Laboratory, Massachusetts Institute of Technology, Cambridge, MA 02139
[g]Broad Institute of Massachusetts Institute of Technology and Harvard University, Cambridge, MA 02142
[h]Department of Brain and Cognitive Sciences, Massachusetts Institute of Technology, Cambridge, MA 02139
[i]Picower Institute for Learning and Memory, Massachusetts Institute of Technology, Cambridge, MA 02139
[j]Institute of Neuroscience, Consiglio Nazionale delle Ricerche, Rozzano, Milan 20089, Italy
[k]Humanitas Clinical and Research Center, Rozzano, Milan 20089, Italy
[l]Kavli Institutefor Neuroscience,Yale University School of Medicine, New Haven, CT 06510

Corresponding author: Pietro De Camilli

Email: pietro.decamilli@yale.edu

This PDF file includes:

Figures S1 to S6
Tables S1
Legends for Movies S1 to S2

Other supplementary materials for this manuscript include the following:

Movies S1 to S2

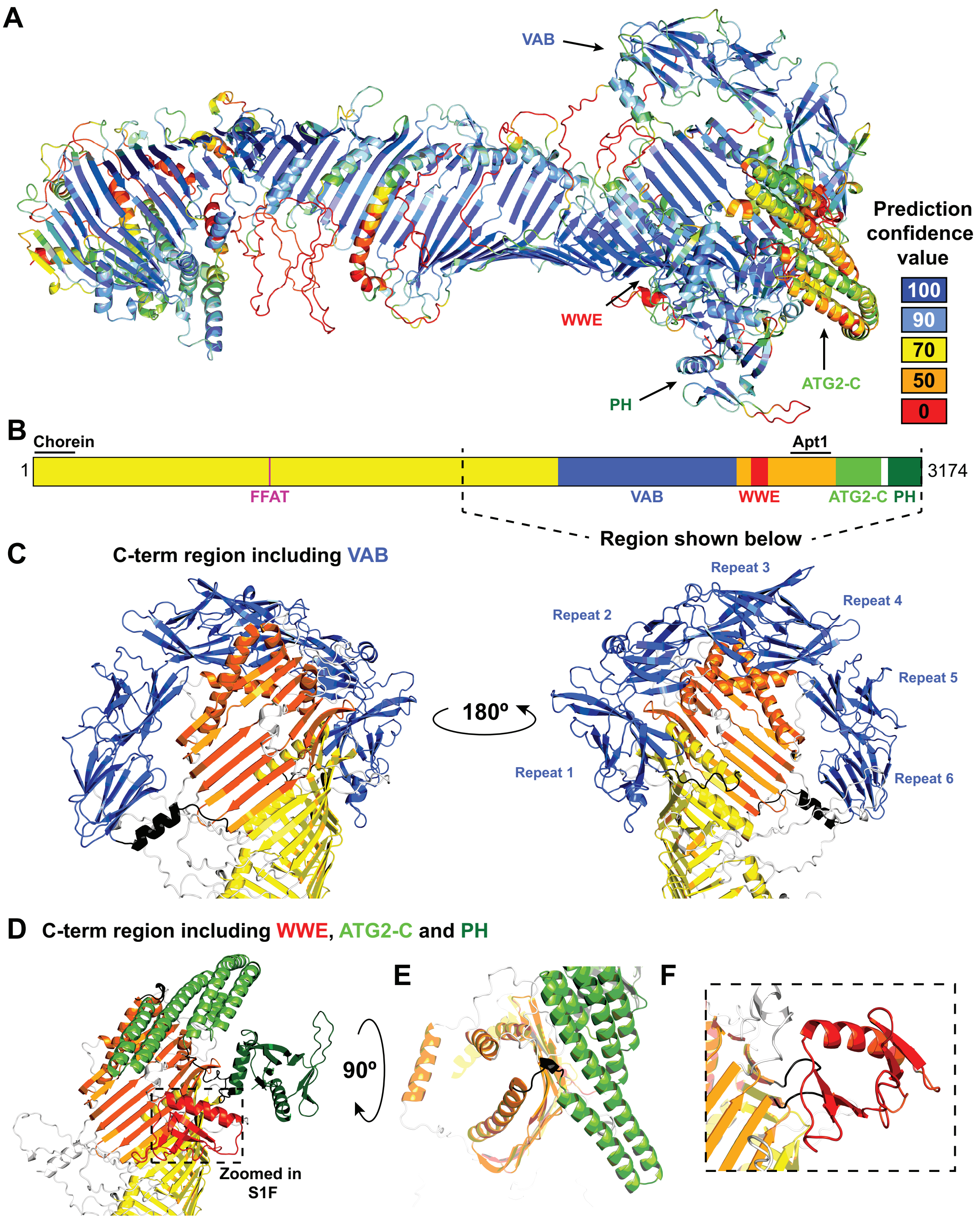

Figure S1. Alphafold predicted VPS13A structure. (A) Full VPS13A structure representation colored by prediction confidence (0-100). Predictions with confidence values ranging from 0-50 should not be interpreted, from 50-70 should be interpreted with caution, from 70-90 the backbone is expected to be modeled well, and from 90-100 are expected to be very close to accurate. Most structured regions of the VPS13A, including the β-sheet groove-harboring rod, the VAB, WWE and PH domains are predicted with good confidence. The ATG2-C domain prediction has lower confidence, probably suggesting some flexibility for this domain, potentially stabilized through its binding to lipids via its hydrophobic surface (see Fig. S5B). (B-F) Structure representations of the

C-terminal region of VPS13A, colored as indicated in the domain cartoon (B). Flexible linkers between structured regions are colored in black. Linkers between the β-sheet rod and the N-terminal and C-terminal ends of the VAB domain are 12 and 18 a.a. long, respectively (C), whereas the linker between the ATG2-C and the PH domains is 25 a.a. long (D). This suggests that the orientation and position shown for these domains represent only one of many possibilities, and that the VAB, ATG2-C and PH domains could be positioned differently when engaged at a membrane. The first helix of the ATG2-C domain is only separated from the core structure of the rod by a short linker of 6 a.a (E), suggesting that the groove harboring rod can make contact with the lipid bilayer when the amphipathic helices in ATG2-C are bound to the bilayer. The WWE domain is an outpocket of the C-terminal end of the β-sheet rod through 4 a.a. long linkers (F). Likely incapable of reaching a membrane, this domain could have a regulatory role via the binding of a ligand.

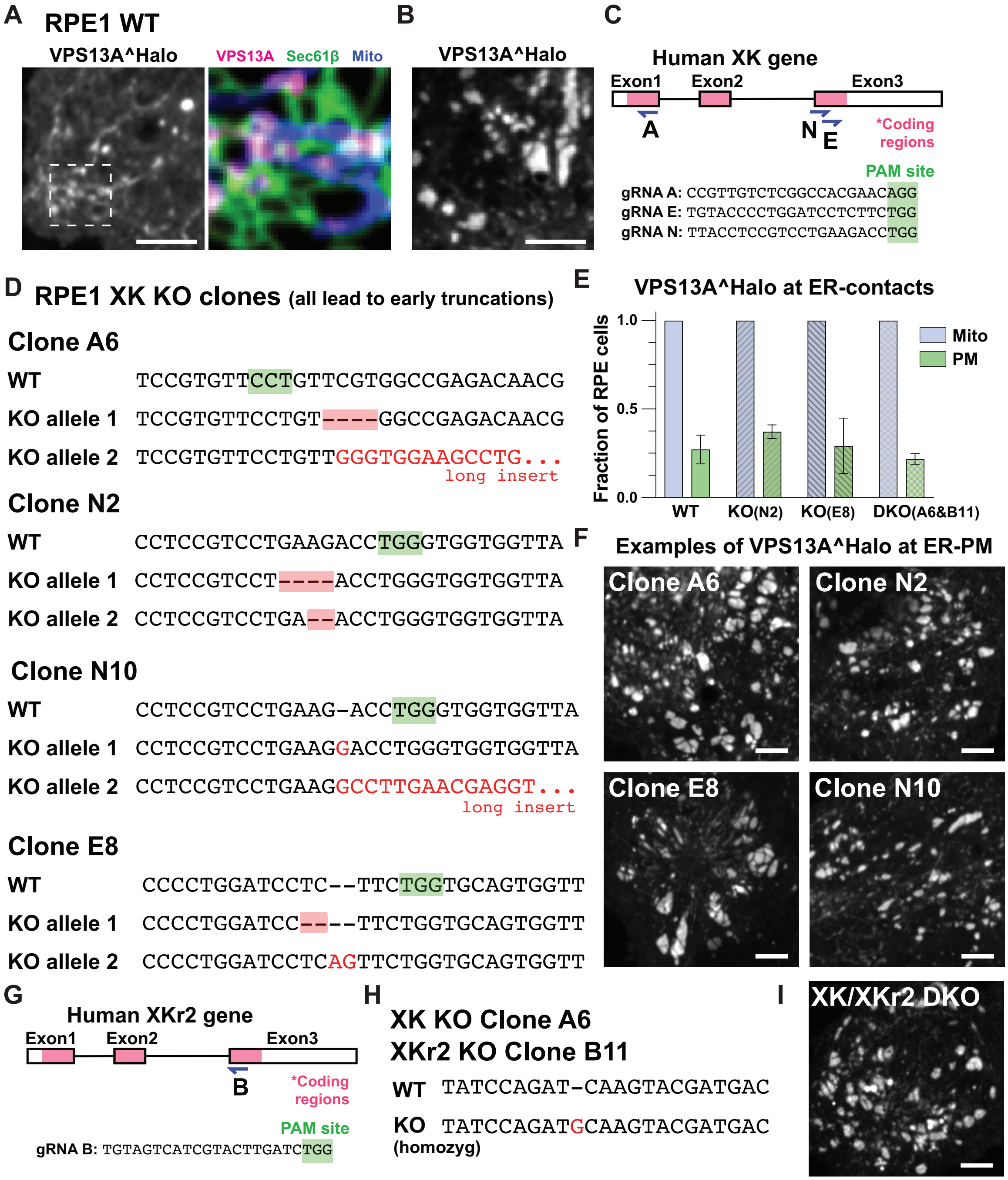

Figure S2. VPS13A can localize to ER-PM contacts in XK KO cells. (A-B) Confocal images of RPE1 cells, expressing VPS13A^Halo showing localization to ER-mitochondria (A) or ER-PM contacts (B). (C) Schematic representation of the XK gene. Three gRNA sequences that target different exons were used. (D) Four different XK KO clonal populations were selected and tested. (E) Fraction of RPE cells expressing VPS13A^Halo that showed a localization to ER-mitochondria and to ER-PM contacts. Data is presented as mean ± SD of a total of 3 experiments. (F) Representative examples of XK KO RPE cells expressing VPS13A^Halo and showing a localization to ER-PM. (G) Schematic representation of the XKR2 gene. (H) Sequence of mutation introduced in XKR2 gene in XK KO cells (clone A6) background. (I) Representative example of XK/XKR2 DKO cell expressing VPS13A^Halo and showing a localization to ER-PM. Scale bars=5μm.

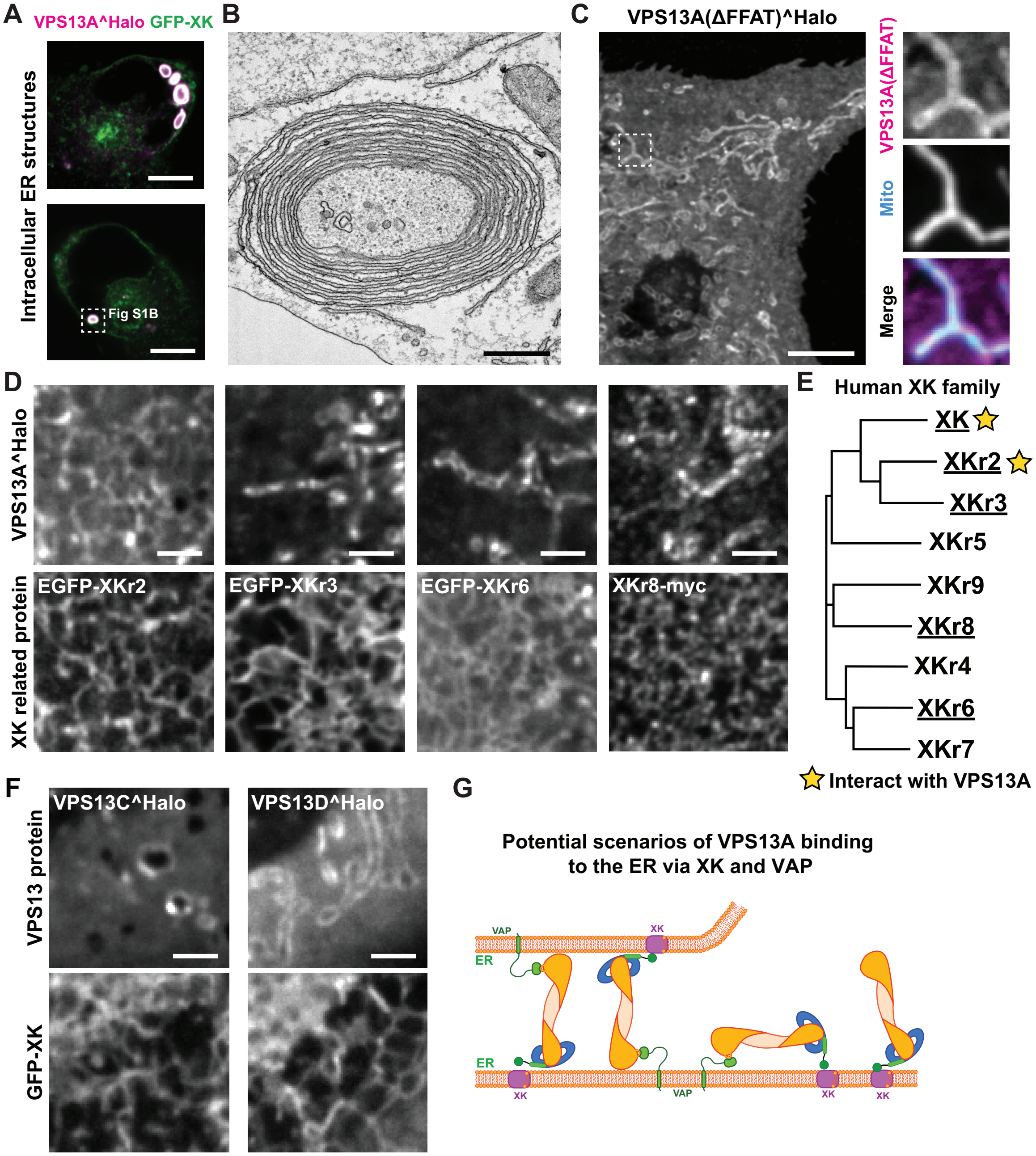

Figure S3. VPS13A interacts with XK and XKr2. (A-B) Binding of VPS13A to XK in the ER. (A) Confocal images of COS7 cells with high enrichment of co-expressed VPS13A^Halo and GFP-XK in round and oval hollow structures that Correlative-Light Electron Microscopy (CLEM) revealed to be circular stack of ER cisternae (B). We speculate that these structures, which resemble OSERs (Organized Smooth ER)(31)) represent appositions of ER membranes generated as shown by the cartoon shown in (G). Note in (G) that the binding of VPS13A does not require VAP. (C) COS7 cell showing localization of VPS13A(ΔFFAT)^Halo to mitochondria when XK is not overexpressed. (D) COS7 cells co-expressing VPS13A^Halo (top images) with XKR2 and other proteins of the XK-related family (bottom images). Top and bottom images shows the same microscopy field. VPS13A^Halo is recruited to the ER by XKR2 but not by XKR3, XKR6 or XKR8. (E) Human XK family tree. The underlined members were tested for interaction as shown in (D). (F) COS7 cells co-expressing GFP-XK with other VPS13 family members, showing that XK does not interfere with VPS13C's localization to endolysosomes or VPS13D's to mitochondria. Top and bottom images shows the same microscopy field. Scale bars=10μm for A and C, 0.5μm for B, 2.5μm for D and F.

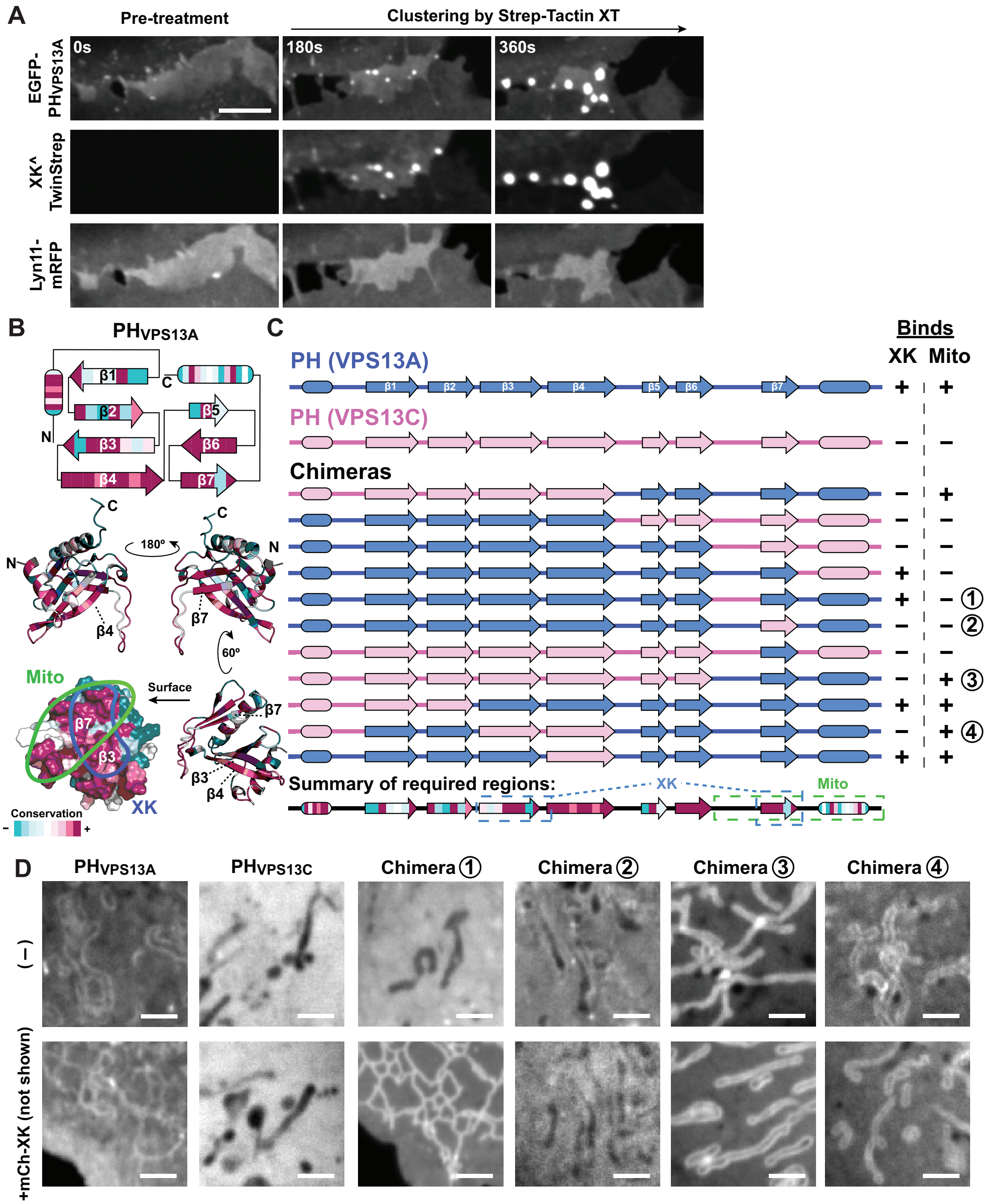

Figure S4. The PH domain of VPS13A interacts with XK and mitochondria via a conserved surface. (A) COS-7 cells co-expressing EGFP-PH$_{VPS13A}$, XK^Twin-Strep and Lyn11-mRFP [a PM marker, (1)] showing that EGFP-PH$_{VPS13A}$, but not Lyn11-mRFP used as a control, co-clusters with XK upon addition of Strep-Tactin XT conjugated to a DY-649 fluorophore (B) Cartoon representing the secondary structure of PH$_{VPS13A}$ (top) and ribbon and surface representations of its tertiary structures predicted by Alphafold (bottom), all colored by conservation among chordates. The surface representation indicates the conserved surface that binds both XK and mitochondria, although by only partially overlapping regions. (C) Diagram indicating all the chimeras between the PH domains of VPS13A and VPS13C that we have tested, and their binding to XK or to

mitochondria. (D) COS7 cells expressing the indicated PH domain construct by itself (top row) or with mCh-XK co-expression (bottom row). Note that chimeras 3 and 4 bind mitochondria with higher affinity than the WT domain. Scale bars=2.5μm.

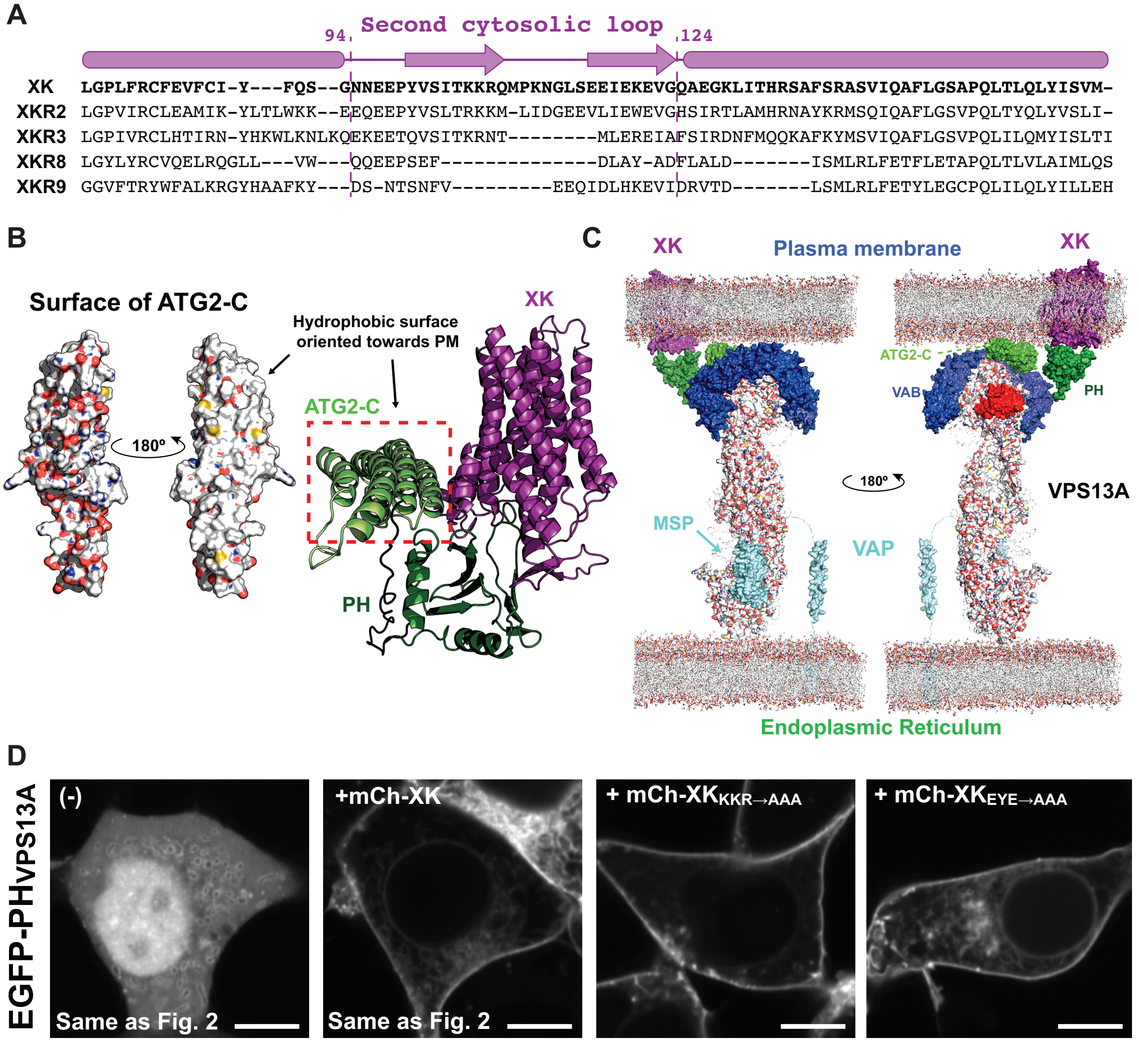

Figure S5. The PH domain of VPS13A interacts with XK and XKR2 via a conserved cytosolic loop. (A) Sequence alignment of the region corresponding to the second cytosolic loop of XK and XKR2/3/8/9. The region interacting with VPS13A is conserved in XKR2, absent in XKR8/9 and partially divergent in XKR3, in agreement with imaging results in Fig. S3D. (B) Alphafold multimer structural prediction of the interaction between XK and the ATG2-C-PH fragment of VPS13A, as shown in Fig. 3F. The ATG2-C domain has a highly hydrophobic surface, shown by white color in the surface representation, which is oriented towards the XK-containing membrane. (C) Schematic of the predicted organization of VPS13A at the ER-PM interface via the interaction with the MSP domain of VAP and with XK. Only one monomer of VAP (a dimeric protein) is shown. (D) HEK293 cells coexpressing the PH domain of VPS13A and the indicated form of XK. The PH domain binds XK in the PM and the ER and is unaffected by the loop 2 and 3 mutations indicated in Fig. 4. Scale bars=5μm.

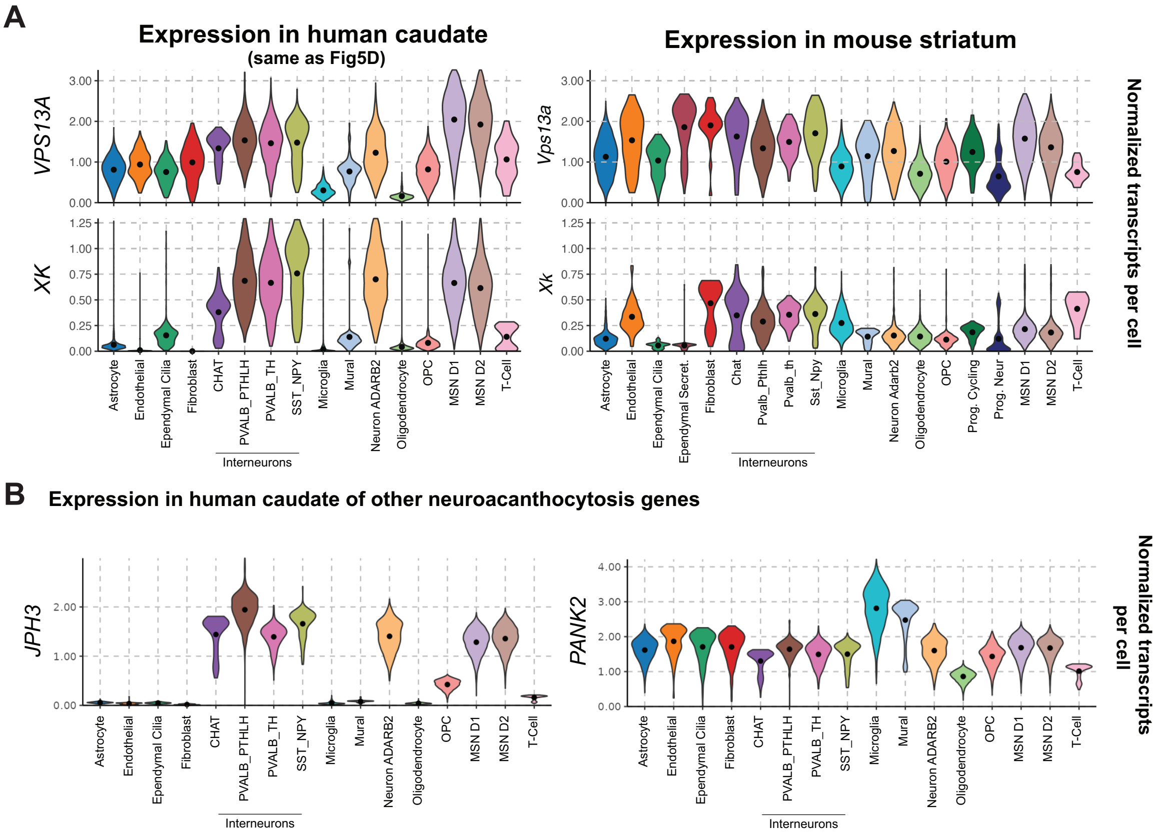

Figure S6. Expression of neuroacanthocytosis genes in human caudate. (A) Expression levels of *VPS13A* and *XK*, as revealed by snRNA-seq, in human (left, same as Fig 5D) and mice (right) striatal cells. *VPS13A* and *XK* are enriched in neurons in human samples whereas their expression is more uniform across cell types in mice. (B) Expression pattern in the human caudate of the two other genes also associated with neuroacanthocytosis syndromes, *JPH3* and *PANK2*. Expression of *JPH3* is restricted to neurons whereas that of *PANK2* is ubiquitous and higher in non-neuronal cell populations.

Table S1. Oligonucleotides used in this study

Construct	Cloning	Templat	Primer sequence	Backbone
**XK constructs**				
XK (no tag)	In-Fusion	**GFP-XK**	CGCTAGCGCTACCGGTccaccATGAAATTCCCGGCCTCG	pEGFP-C1 (EcoRI, AgeI)
			GTCGACTGCAGAATTCTTAAGCAGAGCAGAGATCTTCAGC	
XK^StrepII	PCR amplification	**XK (no tag)**	ATGTATGTGTGCGCACCTCTGT	
			TGCGCACACATACATACCACTTCCTTTTTCGAACTGCGGGTGGCTCCATCCACTACCATAGATGTCAGTCTTGAAAA	
XK^TwinStrep	PCR amplification	**XK^StrepII**	GGAAGTGGTATGTATGTGTGCGC	
			ATACATACCACTTCCTTTCTCAAATTGTGGATGACTCCAAGCAGATCCACCACTTCCACCTCCTGAACCTCCACCTTTTTCGAACTGCGGGTGGC	
mCh-XK	In-Fusion	**GFP-XK**	CTCAAGCTTCGAATTCtATGAAATTCCCGGCCTCG	mCherry-C1 (EcoRI, KpnI)
			GATCCCGGGCCCGCGGTACCTTAAGCAGAGCAGAGATCTTCAGC	
GFP-XK L1->myc	PCR amplification	**GFP-XK**	GTACTGCTGCTGCACCTGC	
			GTGCAGCAGCAGTACCAGATCCTCTTCTGAGATGAGTTTTTGTTCTACGAAGAGAAGCGTGAGCTG[illegible]	
GFP-XK L2->myc	PCR amplification	**GFP-XK**	GCGTTCAGCCGGGCGTCG	
			CGCCCGGCTGAACGCCAGATCCTCTTCTGAGATGAGTTTTTGTTCGCCTGACTGAAAGTAGATGC	
GFP-XK L3->myc	PCR amplification	**GFP-XK**	CTGGCCTATGTCTGTATCTTCC	
			ACAGACATAGGCCAGCAGATCCTCTTCTGAGATGAGTTTTTGTTCATCGTACTTGATTTTGATGGCTAGG	
GFP-XK L4->myc	PCR amplification	**GFP-XK**	AGTAGAGTGGGCACCACCA	
			GGTGCCCACTCTACTCAGATCCTCTTCTGAGATGAGTTTTTGTTCACTGCACCAGAAGAGGATCCA	
GFP-XK L5>myc	PCR amplification	**GFP-XK**	CTGGTGTATTACATGATAAGATTCA	
			CATGTAATACACCAGCAGATCCTCTTCTGAGATGAGTTTTTGTTCTACAGCAGACCAGCAGAACA	
GFP-XK F373*	Quikchange mutagenesis	**GFP-XK**	GAAAAGAGCTTTTTGCAAGGGTGTTAGAACTGATAGAATACAAGCATG	
			CATGCTTGTATTCTATCAGTTCTAACACCCTTGCAAAAAGCTCTTTTC	
mCh-XK (94-115->myc)	PCR amplification	**mCh-XK**	TCAGAGGAGATTGAGAAGGAGGTG	
			CTCAATCTCCTCTGACAGATCCTCTTCTGAGATGAGTTTTTGTTCGCCTGACTGAAAGTAGATGCACAAC	
GFP-XK (EYE->AAA)	PCR amplification	**GFP-XK**	GTCAAAGTGAAGCCTCTGG	
			AGGCTTCACTTTGACAGCTGCAGCATCGTACTTGATTTTGATGGCTAGG	
GFP-XK (KKR->AAA)	PCR amplification	**GFP-XK**	CAAATGCCAAAAAATGGCCTCTCA	
			ATTTTTTGGCATTTGAGCTGCAGCGGTGATACTGACATAAGGCTCTTC	
XK^TwinStrep (KKR->AAA)	PCR amplification	**XK^TwinStrep**	CAAATGCCAAAAAATGGCCTCTCA	
			ATTTTTTGGCATTTGAGCTGCAGCGGTGATACTGACATAAGGCTCTTC	
**VPS13A constructs**				
VPS13A(ΔPH)^Halo	Quikchange Mutagenesis	**VPS13A^Halo**	GTAACATTTGATTTCCAGTCTAATCCCTCAACC	
			AGACCGTACAGGTTGAGGGATTAGACTGGAAAT	
VPS13A(ΔFFAT)^Halo	Quikchange Mutagenesis	**VPS13A^Halo**	GGGACTACATGGTGCATCAATTAATTCCTCCTCTGAATCATCTTC	
			GAAGATGATTCAGAGGAGGAATTAATTGATGCACCATGTAGTCCC	

EGFP-PH(VPS13A)	In-Fusion	**VPS13A^Halo**	CTCAAGCTTCGAATTCtGGGACTGGAAATCAAATGTTACAGG	pEGFP-C1 (EcoRI, KpnI)
			GATCCCGGGCCCGCGGTACCTCAGAGGCTCGGAGAAGG	
VPS13A^Halo^MluI site (used as templat for	Quikchange Mutagenesis	**VPS13A^Halo**	CAAATCTTCCATTTTCCATGACGCGTCTGTAACATTTGATTTCCAGTC	
			GACTGGAAATCAAATGTTACAGACGCGTCATGGAAAATGGAAGATTTG	
VPS13A^Halo (splice B)	In-Fusion	**Ordered cDNA encoding for exon 69 of splice B**	GGAAATCAAATGTTACAGAAAATACAATTCTATAGGGAGTGGATAATGACCCATAGCAGCTCCAGCGACGACGACGATGACGACGATGATGATGACGAGTCTGACCTGAATCATTAAGGATCCTCACAAAGCTACAA	VPS13A^Halo (MluI, BamHI)
**PH VPS13A Chimeras (in same order as listed in Fig S2)**				
EGFP-PH(VPS13C)	In-Fusion	**VPS13C^Halo**	CTCAAGCTTCGAATTCtGATAGACAGGAGTCCGAGGGC	pEGFP-C1
			GATCCCGGGCCCGCGGTACCTCAGGAAGGCAGCTGTGG	
EGFP-PH(N-B4(13C)+B5-C(13A))	In-Fusion	**linearize EGFP-PH(VPS13**	GAATTTACCAAAGAGCCATTCATTG	EGFP-PH(VPS13A)
			AGAATTCGAAGCTTGAGCTCGAG	
		**insert cDNA of PH13C**	CGAGCTCAAGCTTCGAATTCTGATCGACAGGAAAGCGAGGGTTCAGATCTGCTTGAGAATCATATAAAGAAGCTCGAAGGCGAAACATATCGGTACCACTGTGCTATTCCTGGGTCCAAAAAGACTATCCTTATGGTGACAAACAGGAGAGTGTTGTGTATTAAGGAAGTTGAGATTCTCGGATTGATGTGTGTAGATTGGCAGTGTCCCTTCGAGGAATTTACCAAAGAG	
EGFP-PH(N-B4(13A)+B5-C(13C))	In-Fusion	**linearize EGFP-PH(VPS13**	TGAGGTACCGCGGGCCCG	EGFP-PH(VPS13A)
			ATCAAAACTATACTGCCACTCACAC	
		**insert cDNA of PH13C**	CAGTATAGTTTTGATGACTTCGTCTTTCCTCCGTCTGTCTCAGAGAACGTTTTGAAGATCAGCGTTAAAGAACAAGGACTCTTCCATAAAAAGGACTCAGCTAACCAAGGGTGTGTACGCAAGGTTTATCTTAAAGACACAGCCACAGCAGAGCGAGCGTGCAACGCTATTGAGGACGCCCAAAGTACGAGGCAACAACAAAAGTTGATGAAACAGTCATCAGTGCGGCTTCTTAGACCACAGCTCCCGAGTTGAGGTACCGCG[illegible]	
EGFP-PH(N-B6(13A)+B6/B7loop-C(13C))	PCR amplification	**EGFP-PH(VPS13A)**	AAAGAACAAGGACTCTTCCATAAA	
			GAGTCCTTGTTCTTTTGCTTCAATGCGCAATCTTCTCCCATGAACAATGAATGGCTCTTTGGTAAATTCATCAAAACTATACTGCCACTCACAC	
EGFP-PH(N-B7(13A)+C helix(13C))	In-Fusion	**linearize EGFP-PH(VPS13**	TGAGGTACCGCGGGCCCG	EGFP-PH(VPS13A)
			CTTGAAGTTAATTATTTTTCC	
		**insert cDNA of PH13C**	gaaaaataattaacttcaagGACACCGCCACAGCAGAGAGAGCATGTAATGCCATTGAGGATGCACAGTCAACGAGACAGCAGCAAAAATTGATGAAGCAGTCATCAGTGAGACTTCTCAGACCCCAATTGCCATCTTtgaggtaccgcgggcccggg	
EGFP-PH(13A) with B6/B7loop	PCR amplification	**EGFP-PH(VPS13A)**	GGAAAAATAATTAACTTCAAGACCC	
			GTTAATTATTTTTCCAGATGCAGAGTCCTTTTTATGAAAGAGCCCTTGTTCCTTTGCTTCAATGCG[illegible]	
EGFP-PH(13A) with B7 strand 13C	PCR amplification	**EGFP-PH(VPS13A)**	AAGACCCCAGAGGATGCCAG	
			ATCCTCTGGGGTCTTCAAATAAACCTTTCTAACAAACTCTCTGGCATGAAATACAGAC	
EGFP-PH(13C) with B7 strand 13A	PCR amplification	**EGFP-PH(VPS13C)**	ACCGCCACAGCCGAGAGG	
			CTCGGCTGTGGCGGTGGTCTTGAAGTTAATTATTTTTCCAAAGCCCTGATTGGCGGAGTCC	

EGFP-PH(N-B6(13C)+B6/B7loop-C(13A))	PCR amplification	**EGFP-PH(VPS13C)**	TTTGGAAAAATAATTAACTTCAAGA	
			AATTATTTTTCCAAACTCTCTGGCATGAAATACAGACTTCACTCGTTCCTTTGCGCTGATCTTCAGCACGTTCT	
EGFP-PH(N-B2(13C)+B3-C(13A))	PCR amplification	**EGFP-PH(N-B4(13C)+B5-C(13A))**	GAATTTACCAAAGAGCCATTCATTG	
			CTCTTTGGTAAATTCATCAAAACTATACTGCCACTCACACGTGAGTTGTCCAAATGTTCCCTTTGTTACAAACAACACTCTCCTGTTTGTCACCATAAGG	
EGFP-PH(13A) with B3B4 strands 13C	PCR amplification	**EGFP-PH(N-B4(13C)+B5-C(13A))**	AACAGGAGAGTGTTGTGTATTAAGG	
			CAACACTCTCCTGTTGGTTATCATTAGCATATCTGTCTTATTGATCATGACATGGGTAAAATACCGATATGTTTCGCCTTCGAGC	
EGFP-PH(13A) with B4 strand 13C	PCR amplification	**EGFP-PH(VPS13A)**	AGTTTTGATGAATTTACCAAAGAGC	
			AAATTCATCAAAACTACACTGCCAGTCCACGCACATCAGTCCAAATGTTCCCTTTGTTACAAAC	
**Other Constructs**				
EGFP-XKr3	In-Fusion	**Xkr3 (Genscript)**	CTCAAGCTTCGAATTCtATGGAGACAGTGTTTGAAGAGATGG	pEGFP-C1 (EcoRI, KpnI)
			GATCCCGGGCCCGCGGTACCttaTGAACATGTCATACTTTTTCTG	
EGFP-XKr6	In-Fusion	**Xkr6 (Origene)**	CTCAAGCTTCGAATTCtATGGCGGCGAAATCCGATGG	
			GATCCCGGGCCCGCGGTACCTTAGAGTGAAGACTCATACTGTAGC	
px459-XK gRNA AA	Hi-Fi	gRNA AA	ATCTTGTGGAAAGGACGAAACACCGCCGTTGTCTCGGCCACGAACGTTTTAGAGCTAGAAATAGCA	px459 (BbsI)
px459-XK gRNA AE		gRNA AE	ATCTTGTGGAAAGGACGAAACACCGTGTACCCCTGGATCCTCTTCGTTTTAGAGCTAGAAATAGCA	
px459-XK gRNA AN		gRNA AN	ATCTTGTGGAAAGGACGAAACACCGTTACCTCCGTCCTGAAGACCGTTTTAGAGCTAGAAATAGCA	

**Primers for sequencing**	
XK genomic DNA exon 1	GGTTTGGGGCTGGGCAT
	AGGTGCAGCAGCAGTACG
XK genomic DNA exon 3	GTGAAGCCTCTGGCCTATGT
	CCCAGCAAAAACACCTGAGC

Movie legends

Movie 1. VPS13A is a ~22 nm long rod with a hydrophobic cavity. Rotating VPS13A structure in ribbon and surface representation. The ribbon representation is colored as in Figure 1A and the surface representation by element: carbon in white, nitrogen in blue (positive charges) and oxygen in red (negative charges).

Movie 2. The PH domain of VPS13A interacts with XK at the PM. COS-7 cell co-expressing EGFP-PH_{VPS13A} and XK^Twin-Strep showing co-clustering of both proteins upon addition of Strep-Tactin XT conjugated to a DY-649 fluorophore at time=0s. Scale bar=5μm.

SI References

1. O. Idevall-Hagren, E. J. Dickson, B. Hille, D. K. Toomre, P. De Camilli, Optogenetic control of phosphoinositide metabolism. *P Natl Acad Sci Usa* 109, E2316-23 (2012).
